# American Medical Association

**Published:** 1850-03

**Authors:** Daniel Drake, D. P. Strader

**Affiliations:** Chairman; Cincinnati; Secretary; Cincinnati


AMERICAN MEDICAL ASSOCIATION.—THIRD ANNUAL MEETING.

In consequence of a delay, up to the present time, in the distribution of the Transactions of the second meeting of the Association, held at Boston in the month of May, 1849, the *Standing Committee of Arrangements*, appointed at that time, deem it expedient to give public notice, through the medical periodical press, that the next meeting will be held in Cincinnati, on Thursday, the 7th of May, ensuing; and, at the same time, they wish to make known to the physicians, who reside in portions of the United States from which few or no delegates have yet been sent, the terms of membership. This they will do, by copying a part of the second article of the Constitution:

"*The Delegates* shall receive the appointment from permanently organized medical societies, medical colleges, hospitals, lunatic asylums, and other permanently organized medical institutions of good standing in the United States. Each delegate shall hold his appointment for one year, and until another is appointed to succeed him, and shall participate in all the business and affairs of the association.

"Each local society shall have the privilege of sending to the association one delegate for every ten of its regular resident members, and one for every additional fraction of more than half of this number. The faculty of every regularly constituted medical college or chartered school of medicine, shall have the privilege of sending two delegates. The professional staff of every chartered or municipal hospital containing a hundred inmates or more, shall have the privilege of sending two delegates; and every other permanently organized medical institution of good standing, shall have the privilege of sending one delegate.

"*The Members by Invitation* shall consist of practitioners of reputable standing, from sections of the United States not otherwise represented at the meeting. They shall receive their appointment by invitation of the meeting after an introduction from any of the members present, or from any of the absent permanent members. They shall hold their connection with the association until the close of the annual session at which they are received; and shall be entitled to participate in all its affairs, as in the case of delegates.

"*The Permanent Members* shall consist of all those who have served in the capacity of delegates, and such other members as may receive the appointment by unanimous vote.

"Permanent members shall at all times be entitled to attend the meetings, and participate in the affairs of the association, so long as they shall continue to conform to its regulations, but without the right of voting; and when not in attendance, they shall be authorized to grant letters of introduction to reputable practitioners of medicine residing in their vicinity, who may wish to participate in the business of the meetings, as provided for members by invitation."

The Committee desire, still farther, to give notice that it is their duty to receive and present the reports of any of the standing Committees, whose members cannot attend the meeting; and all communications on scientific subjects, which gentlemen, not members of the Association, may desire to lay before it.

It is likewise, their duty to examine the credentials of delegates and register their names, which it is desirable should, as far as possible, be done before the meeting of the Association, for which purpose the Committee will meet on the preceding day.

In conclusion, the Committee indulge the hope, that this first meeting in the interior of the continent, will be well attended by its physicians, and all who cultivate the sciences auxiliary to medicine; that no society, college or hospital will remain unrepresented; and that many distinguished physicians, not appointed as delegates, will attend and become members by a vote of the Association.

Daniel Drake, M. D., *Chairman.*

D. P. Strader, M. D., *Secretary.*

*Cincinnati*, Jan. 31, 1850. *Ibid.*

